# COVID-19 Symptomatic Newborns with Possible Postpartum Transmission of SARS-CoV-2

**DOI:** 10.1155/2022/7394175

**Published:** 2022-09-27

**Authors:** Mohammad Hosein Ataee Nakhaei, Sahar Safapour Moghadam, Saeedeh Yaghoubi

**Affiliations:** ^1^Department of Pediatrics, Neonatal Intensive Care Unit (NICU), Faculty of Medicine, Zahedan University of Medical Sciences, Zahedan, Iran; ^2^Department of Pediatrics, Faculty of Medicine, Zahedan University of Medical Sciences, Zahedan, Iran; ^3^Zahedan University of Medical Sciences, Zahedan, Iran

## Abstract

Coronavirus disease 2019 (COVID-19) infection, which was initially reported in Wuhan, China, in December 2019, had a rapid spread throughout the world becoming a new global crisis. Today, very little is known about neonatal COVID-19 infection. Herein, we tried to define the clinical and demographic characteristics, risk factors, and laboratory and imagining findings of neonates who tested positive for COVID-19 and were admitted to the NICU (neonatal intensive care unit) of Ali Ibn Abi Taleb Hospital, Zahedan, Iran, from June 2021 to July 2021. All full-term and premature neonates diagnosed with COVID-19 were included in the study. Their ages ranged from 1 to 21 days at admission, including 6 boys and 4 girls. The medical records of mother-baby dyads were reviewed. All mothers, except for one, were negative for COVID-19 infection. The most frequent findings in the neonates were fever, poor feeding, respiratory distress, cough, hypoxemia, and drooling. Broad-spectrum antibiotics were administered as routine. All neonates, except for one, needed respiratory support, and intratracheal surfactant was administered for three newborns. Three neonates with severe disorders died during the study period.

## 1. Introduction

Coronavirus disease 2019 (COVID-19) infection, which was initially reported in Wuhan, China, in December 2019, had a rapid spread throughout the world becoming a new global crisis. COVID-19 affects all age groups, but the prevalence and severity of infection in children are less than in adults [1, 2]. Moreover, the severity of symptoms among children is milder than among adults. Systematic reviews on SARS-CoV-2 infection in neonates have reported that the infection cannot transmit vertically, yet further investigation seems essential in this respect [3–5].

### 1.1. Clinical Findings of COVID-19 in Neonates

The clinical manifestation of COVID-19 in neonates ranges from asymptomatic disease to severe respiratory distress. Its clinical symptoms in the pediatric age group consist of fever, dry cough, sputum, nasal discharge, sore throat, rhinorrhea, and stuffy nose. Some patients have gastrointestinal manifestations such as diarrhea, vomiting, and poor feeding (4). The most common clinical presentations among symptomatic neonates are respiratory distress, fever, and poor feeding [6]. Some studies have reported a more severe form of the disease in infants and children under 1 year than in older children [6]. Diagnosis of the disease is based on medical history and thorough laboratory tests. The real-time reverse transcriptase polymerase chain reaction (PCR) assay of nasopharyngeal swabs is the *gold standard* for confirmation of COVID-19 infection.

### 1.2. Treatment

There is no known specific treatment for SARS-CoV-2 in the neonatal population. Supportive therapy including maintenance of the water-electrolyte hemostasis, nutrition, acid-base balance, and respiratory supply as needed is recommended. Furthermore, intratracheal surfactant administration, antibiotics, and mechanical ventilation should be applied based on the individual's need [6]. In this case series, the demographic, epidemiologic, and clinical features of the neonates hospitalized in the NICU of Ali Ibn Abi Taleb Hospital in Zahedan were investigated. The severe acute respiratory syndrome coronavirus 2 (SARS-CoV-2) real-time reverse transcriptase PCR test was performed using nasopharyngeal swab samples in all suspected neonates and their mothers. Positive cases of COVID-19 infection were included in this study. Data were collected from June 2021 to July 2021.

### 1.3. Case Presentation

In the present study, 10 neonates with suspected neonatal sepsis were admitted to the NICU of Ali Ibn Abi Taleb Hospital, Zahedan, Iran, from June 2021 to July 2021. All 10 individuals had a positive SARS-CoV-2 PCR test. Demographic characteristics, clinical signs, laboratory tests, and treatment measures of the studied neonates are presented in Table 1. The mean gestational age and birth body weight of the newborns were 36.5 weeks (range: 30–40) and 2508 g (range: 1280–3900 g), respectively. Most cases (60%) were male. The male-to-female ratio of hospitalized cases was 1:5. Their mean Apgar score was greater than 6 at 5 min. Among our studied patients, except for one case, all mothers were negative for SARS-CoV-2. Moreover, six cases had a positive PCR test in their father or other family members. However, the possibility that third parties, including hospital staff and nearby people, may have infected the infants cannot be excluded. Four of these infants were born by cesarean section, and 6 others, including the neonate who had a positive history of COVID-19 in her mother, were born by normal vaginal delivery (NVD). One preterm infant was admitted at delivery, and the rest were admitted a few days after birth. The most common symptoms included fever, respiratory distress, poor feeding, and restlessness. Less frequent symptoms were drooling and apnea. Four of the studied neonates had concomitant disorders including congenital heart disease (ventricular septal defect and symptomatic patent ductus arteriosus) and hydrocephaly. Positive findings in the examination of these neonates included reduced neonatal reflexes, hypotonia, cyanosis, and respiratory distress. Because of the COVID-19 pandemic, a nasopharyngeal swab RT-PCR testing for SARS-CoV-2 was requested for all neonates and their mothers, besides the routine neonatal sepsis workup. During hospitalization, all neonates received broad-spectrum antibiotics; three of the neonates were treated with surfactants, and 9 cases needed respiratory support, including tracheal intubation (4 cases), nasal CPAP (2 cases), and oxygen therapy with an oxy hood (3 cases). Only one infant did not require any respiratory support. Moreover, intracranial ultrasonography on day one and cardiac consultation on day three were performed for all cases. Eventually, three patients died due to multisystem failure, and the others were discharged without any complications.

## 2. Discussion

We studied 10 neonates hospitalized with findings indicative of neonatal sepsis and subsequent positive RT-PCR testing for COVID-19. The most common findings in our patients were respiratory distress and fever (>38°C). Among the reported symptomatic pediatric cases, the most common clinical presentation has been fever in infants above one month of age [7], whereas respiratory distress, fever, and poor feeding were the most common findings in neonates [8].

In the study by Tabatabai, fever was reported in most of the studied patients [9]. In a survey by De Bernardo et al., the main findings at onset were fever, vomiting, cough or shortness of breath, diarrhea, lethargy, and respiratory difficulty. Other rare findings included cyanosis, feeding intolerance, hyperpnea, mild intercostal retractions, mottling, sneezing, nasal stuffiness, and paroxysmal episodes [10].

In the current study, all cases, except for one, required respiratory support; however, none of the studied neonates had significant gastrointestinal symptoms, except for drooling.

The laboratory findings reported in pediatric SARS-CoV-2 infection are inconsistent with those in adult cases. The main laboratory findings include leukocytosis, lymphopenia, elevated creatine phosphokinase, liver enzymes, C-reactive protein, and/or procalcitonin levels [7]. Rare cases of disseminated intravascular coagulation (DIC) and multiorgan dysfunction resulting in neonatal death have also been reported [11]. In our study, lymphopenia (<3000 × 1000/mm^3^ in infants) was noted in 4 cases out of 10, and all cases had increased CRP (normal range< 5 mg/L) levels.

The abnormal radiological findings reported by Ilaria Liguoro et al. [7] and by Tabatabaei [9] were detected in less than half of our cases, yet specific lesions were not so common. In a systematic review by Ilaria Liguoro et al., less than half of the neonates (48%) had abnormal findings in chest radiography, whereas only one of our cases had an abnormal CXR, including hyperinflation and stomach dilation. A rare case of an infant with myocarditis and a positive COVID-19 respiratory distress test was reported by Amiraskari et al. [12]. Accordingly, four of our patients had congenital heart disease; 2 cases had VSD, 1 had a significant PDA, and another one had a large PFO. This system is for classifying SARS-CoV-2 vertical transmission. The evaluation of the frequency and timing of vertical transmission of SARS-CoV-2 raises some methodological challenges. The virus is primarily transmitted through the respiratory route, and respiratory samples are primarily used for diagnosis, making it difficult to differentiate in utero or intrapartum transmission from the postnatal transmission. Based on a review of other congenital infections and what is known about modes of transmission and diagnosis of SARS-CoV-2, several issues were considered during the development of the classification system, as described below. Definition and timing of maternal infection: maternal infection is defined as per WHO COVID-19 case definitions. There are only limited data on fetal outcomes of women who had SARS-CoV-2 in early pregnancy. There are some case reports of first or early second-trimester pregnancy loss in women with symptomatic COVID-19, but several of these cases lack data on SARS-CoV-2 detection in the corresponding placenta and/or fetus. The rate of spontaneous abortion (<13 weeks gestation) in women with SARS-CoV-2 infection in the first trimester does not appear to be increased when compared to women with ongoing pregnancies (11% (11/100) vs 9.3% (12/125)). The few reports of amniocentesis in women with recent/recovered SARS-CoV-2 infection have not shown evidence of amniotic fluid infection. However, because the potential for in utero transmission with maternal infection in early pregnancy is unknown, for the diagnosis of in utero infection, documented SARS-CoV-2 maternal infection at any time during pregnancy is considered appropriate; for diagnosis of intrapartum and postnatal transmission, the maternal infection must be diagnosed near the time of childbirth from 14 days before 2 days after birth. The main reasons for the positive COVID-19 result are the following:

1. Laboratory errors and unknown reactions (for example, a virus reacts differently to a COVID-19 virus in the face of a test)
2. Simultaneous infectious diseases and increase in the number of viruses in the body (very severe colds and underlying respiratory diseases, etc.)
3. Wrong sampling
4. Medical record errors [13].

Given the small sample size of this study, it is not possible to generalize our results to all neonates. However, the findings on this new infection in the neonatal population can be strongly considered and further investigated in future studies. The main limitation of the current study was the absence of a chest CT scan in the recruited patients.

## 3. Conclusion

Our findings highlight the importance of performing COVID-19 test in all newborns admitted to the NICU, irrespective of the mother's history, as they remain at risk of contracting this infection in the neonatal period and the long-term consequences are still unknown.

Although the vertical transmission of COVID-19 is less likely, the horizontal spread of this novel infection from asymptomatic parents, siblings, family members, or healthcare providers can be a major source of infection in the studied age group.

Nevertheless, there are treatment options for neonatal COVID-19 infection except for supportive care and symptomatic treatment of complications.

## Figures and Tables

**Table 1:** Demographic and characteristic data of neonates infected with COVID-19.

Demographic data	Case 1	Case 2	Case 3	Case 4	Case 5	Case 6	Case 7	Case 8	Case 9	Case 10
Gestational age	37 w	39 w	30 w	37 w	37 w	39 w	39 w	40 w	30 w	37 w

Gender	Male	Female	Female	Male	Female	Male	Male	Female	Male	Male

Type of birth	C/S	NVD	C/S	C/S	NVD	NVD	NVD	NVD	C/S	NVD

Birth weight (g)	3900	2600	1280	2200	3200	2000	3100	3200	1600	2000

APGAR scores at 1 and 5 min	(8–9)	(8–9)	(2–6)	(8–9)	(9–10)	(9–10)	(9–10)	(9–10)	(5–8)	(8–9)

Concomitant disorders	VSD	−	PDA	Hydrocephaly	−	−	−	−	−	−

Postnatal age at admission, day	21 d	10 d	At birth	15 d	18 d	3 d	11 d	15 d	15 d	8 d

Length stay, day	8 d	4 d	21 d	10 d	4 d	6 d	7 d	6 d	11 d	10 d

Positive maternal history of COVID-19	−	−	+	−	−	−	−	−	−	−

Positive family history of COVID-19	+	+	+	−	+	−	+	+	−	−

Clinical manifestation										

Temperature	38.3	36.6	36.7	38.1	37.9	36.9	38.7	38	36	38.5

Respiratory distress (tachypnea, grunting, nasal flaring)	+	+	+	+	+	+	−	−	+	+

GI symptoms	−	−	−	−	−	−	−	−	−	−

Drooling	+	−	−	+	+	+	−	−	+	−

Seizure	−	−	−	−	−	−	−	−	−	−

Laboratory findings										

WBC (×1000/mm^3^)	7.1	6	11.8	12.3	12.5	13.9	14.7	2.1	NA	11.1

ALC (%)	34	46.6	42.8	18.8	59.6	27.6	47.3	25.2	NA	38.8

ALC (cells/mm^3^)	2414	2796	5050	2312	7450	3836	6953	529	NA	4307

Absolute PMN count (%)	60	44.3	48.8	72.8	−	−	−	51.5	−	56

PLT (×1000/mm^3^)	194	111	337	143	323	53	330	238	NA	73

CRP (mg/L)	5	5	96	8	95	5	96	5	5	7

LDH (IU/L)	879	630	972	442	771	999	613	NA	564	953

Blood group	B^+^	O^+^	O^+^	NA	A^+^	A^+^	O^+^	NA	O^−^	B^−^

Imaging										

CXR	Over inflammation + stomach dilation	NL	NL	NL	NL	NL	NL	NL	NL	NL

Brain sonography	NL	NL	NL	Ventriculomegaly	NL	NL	NL	NL	NL	NL

Cardiovascular	Large VSD	NL	PDA	Large ASD + large VSD + PH	Large PFO + ASD + MI LD TR	NL	NL	NL	NL	NL

Respiratory support										

Surfactant treatment	+	−	+	−	−	−	−	−	+	−

Mechanical ventilation	+	+	+	−	−	+	−	−	−	−

nCPAP	−	−	−	−	−	−	−	−	+	+

Oxygen hood	−	−	−	+	+	−	−	+	−	−

No respiratory support	−	−	−	−	−	+	−	−	−	−

Outcomes of the neonates										

Dead	+	+	−	−	−	+	−	−	−	−

Alive	−	−	+	+	+	−	+	+	+	+

Outcomes of the mother										

Dead	−	−	+	−	−	−	−	−	−	−

Alive	+	+	−	+	+	+	+	+	+	+
